# The health digital twin to tackle cardiovascular disease—a review of an emerging interdisciplinary field

**DOI:** 10.1038/s41746-022-00640-7

**Published:** 2022-08-26

**Authors:** Genevieve Coorey, Gemma A. Figtree, David F. Fletcher, Victoria J. Snelson, Stephen Thomas Vernon, David Winlaw, Stuart M. Grieve, Alistair McEwan, Jean Yee Hwa Yang, Pierre Qian, Kieran O’Brien, Jessica Orchard, Jinman Kim, Sanjay Patel, Julie Redfern

**Affiliations:** 1 University of Sydney, Faculty of Medicine and Health, Sydney, NSW Australia; 2 The George Institute for Global Health, Sydney, NSW Australia; 3 Kolling Institute of Medical Research, Royal North Shore Hospital, Sydney, NSW Australia; 4 University of Sydney, School of Chemical and Biomolecular Engineering, Sydney, NSW Australia; 5 University of Sydney, Charles Perkins Centre, Sydney, NSW Australia; 6 Department of Cardiology, Royal North Shore Hospital, Sydney, NSW Australia; 7 Cincinnati Children’s Hospital Medical Cente, Cincinnati, OH USA; 8 The University of Sydney, School of Biomedical Engineering, Sydney, NSW Australia; 9 Westmead Applied Research Centre, Westmead Hospital, Sydney, NSW Australia; 10 Siemens Healthcare Pty Ltd; and Centre for Advanced Imaging, University of Queensland, Brisbane, QLD Australia; 11 University of Sydney, School of Computer Science, Sydney, NSW Australia; 12 Royal Prince Alfred Hospital, Sydney, NSW Australia; 13 Heart Research Institute, Sydney, NSW Australia

**Keywords:** Predictive medicine, Computational platforms and environments

## Abstract

Potential benefits of precision medicine in cardiovascular disease (CVD) include more accurate phenotyping of individual patients with the same condition or presentation, using multiple clinical, imaging, molecular and other variables to guide diagnosis and treatment. An approach to realising this potential is the digital twin concept, whereby a virtual representation of a patient is constructed and receives real-time updates of a range of data variables in order to predict disease and optimise treatment selection for the real-life patient. We explored the term digital twin, its defining concepts, the challenges as an emerging field, and potentially important applications in CVD. A mapping review was undertaken using a systematic search of peer-reviewed literature. Industry-based participants and patent applications were identified through web-based sources. Searches of Compendex, EMBASE, Medline, ProQuest and Scopus databases yielded 88 papers related to cardiovascular conditions (28%, *n* = 25), non-cardiovascular conditions (41%, *n* = 36), and general aspects of the health digital twin (31%, *n* = 27). Fifteen companies with a commercial interest in health digital twin or simulation modelling had products focused on CVD. The patent search identified 18 applications from 11 applicants, of which 73% were companies and 27% were universities. Three applicants had cardiac-related inventions. For CVD, digital twin research within industry and academia is recent, interdisciplinary, and established globally. Overall, the applications were numerical simulation models, although precursor models exist for the real-time cyber-physical system characteristic of a true digital twin. Implementation challenges include ethical constraints and clinical barriers to the adoption of decision tools derived from artificial intelligence systems.

## Introduction

Cardiovascular disease (CVD) accounts for approximately one-third of all deaths globally and is the leading cause of disability-adjusted life years—the years lived with disability and years of life lost due to premature death^1^. Further, ischaemic heart disease (IHD) surpasses all other types of CVD as a cause of premature mortality, with access to, and adoption of, proven treatments being context-specific^1^. Attention to accurate and personalised risk assessment with tailored prevention treatments remains imperative. Currently, the risk of CVD and IHD is estimated using risk algorithms incorporating a small number of traditional risk factors. However, the substantial number of events occurring in individuals considered low risk by traditional algorithms^2^ and therapy resilience in those with risk factors^3^ highlight that many questions remain to optimise the effective use of preventative medicines, devices, and other therapies, from both health and economic perspectives.

Precision or personalised medicine is an evolving field worldwide and seeks to more accurately phenotype individual patients with the same condition or presentation, allowing tailored screening, diagnostics, and treatment^4^. Broad application of this concept has been facilitated by biological databases (such as the genome sequence)^4^ and use of bio- and other markers to stratify patients for more targeted therapy^5^. For years, ‘omics’ technologies have measured the activities of thousands of genes (transcriptomics), proteins (proteomics) or other molecular features simultaneously from a mixed collection of cells that generate high-dimensional complex data now termed ‘omics’ data, which advance understanding of the genotype-to-phenotype relationship^6^. The important premise is that genetic, microbial, proteomic, metabolic, clinical, and behavioural pathways characterise patients and their health^4^. Advanced computational techniques for large data sets may overcome this inherent variability between individuals for more precise clinical decision-making and choice of interventions^4^. An approach to realising the possibilities of precision medicine is the concept of the digital twin, whereby patient-specific therapy is based on using a virtual replica (the digital twin) to predict treatment outcome and to personalise prognosis for a patient (the real-life twin).

The health digital twin has its origins in the established industry practice of creating virtual models of physical systems or assets in the field to enable planning decisions, risk assessment, testing, and to anticipate maintenance^7^. Such feedback systems use new technologies, such as cloud computing, 5G communication networks, and prediction software, to enable a three-way convergence of the virtual model, the physical asset, and the real-time data acquisition and exchange between them^7^, which occurs via networking devices or sensors located in each twin^8^. Analytical algorithms extract, store, and integrate the data acquired from multiple sources to detect changes, trends and patterns, predict and diagnose failures, test alternative decisions, and overall optimise the performance of the real-life asset^8^. For this reason, twinning a process or an object offers cost and time-saving advantages over a model or simulation technique, and is routinely used in rail, road, and maritime transport, supply chain and plant operations, and civil engineering^8,9^. The manufacturing sector, for example, uses virtual twins of machinery or factory equipment^8^; also modern consumer products, such as smart cars^9^. An aircraft’s digital twin is critical to maintaining its structural and mechanical health over its lifetime^8^. At the healthcare facility and department level, testable scenarios based on real-time data inputs to a mirrored system are proposed to improve processes for staff allocation, visitor/patient flow, waiting time, equipment and other internal resource provision, emergency vehicle access and other service-related operations^10^.

In a similar way, and drawing also on origins within the human genome project^4^, an individual health digital twin receives a variety of data parameters to assist decision-making and predictive evaluations for a real-life patient. The overall construct is centred on a population-based databank comprising two key types of data. Firstly, deep phenotyping as sourced from electronic health records, biological, clinical, genetic, molecular, and imaging data. Secondly, the phenotyping of real-world data from the person’s environment, using mobile data sensors and wearable devices^11^. Assimilating these continuously acquired, multi-source data into clinically meaningful knowledge occurs through an automated, iterative process of data pre-processing, data mining, and data integration, that produces more useful information than is provided by any single data source^8,12^. In the cardiology context, these phenotypic data for a digital twin are analysed in a predictive framework comprising combined statistical and mechanistic modelling that enables reasoning in the twin^13^. From within the population-based databank, a real-life patient has a digital twin selected that represents the average characteristics of its closest cluster group^11^. The outcome of virtual interventions subsequently given to the real-life patient then feeds back into the databank to both modify the twin and add to the population data pool^11^. This dynamic loop is crucial to expanding the databank and ensuring its diverse physiological and demographic make-up. The paradigm draws on bioengineering and computer sciences to aggregate and analyse information from large patient cohorts.

Given the significant potential for the digital twin principle to empower CVD research, fuller understanding of its scope will be beneficial. Therefore, the aims of this review were to describe the research designated ‘digital twin’, with a focus on uses of the term and concept within CVD-related research; to summarise the key concepts; to identify the disciplines contributing to the field; and to describe the emerging challenges for the progress of digital twin research within the wider context of precision medicine.

## Methods

### Study design

We undertook a mapping review, modified to accommodate the broad questions of interest. A mapping review is exploratory in nature for the purpose of providing an overview about a topic, or determining the volume and nature of literature within a field^14^. Elements of systematic review methodology ensured a transparent and replicable search process, with adaptations in part because the focus was on the breadth of information and comprised heterogeneous research and non-research material. Hence, a systematic approach was taken to literature searching, but unlike a systematic review a mapping review excludes critical appraisal of the methodological quality of the literature and evidence synthesis^15^.

### Database search strategy

Eligible papers from the peer-reviewed literature were identified from Compendex, EMBASE, Medline, ProQuest and Scopus databases. The search strategy was then adapted to each database. (Supplementary Note 1) Reference lists, including the results of citation chaining (a function within the Scopus database) were hand-searched to identify further publications or grey literature. Papers were included if they were published in English and if they were a research/experimental report, commentary, narrative, descriptive paper, or a book chapter. Letters, editorials, media articles and conference abstracts were excluded. No limitations were set for the publication date.

### Other data sources

Names of companies were identified from article reference lists and internet searching. Websites were then reviewed to characterise the industry participants and their products in the CVD and non-CVD digital twin fields. Publicly available patent information was searched on the websites of the Canadian Intellectual Property Office (www.cipo.ic.gc.ca); the European Patent Office (www.epo.org/index.html); the United Kingdom Intellectual Property Office (www.gov.uk/government/organisations/intellectual-property-office); the United States Patent and Trademark Office (https://appft.uspto.gov/); and the World Intellectual Property Organization (https://patentscope.wipo.int/search/en/search.jsf).

### Charting the data

The results that were charted (the step analogous to data extraction in a systematic review^14,15^) were limited to five fields: publication year; first author country; article type; the condition targeted and first author academic discipline. Additional information from the CVD-related papers was the purpose of the project; key concept or methodology; status of the digital twin model; and the databank used or created. Data from industry-related websites included the target condition(s); the digital twin product or databank; and availability of the product or development status. Patents were searched for the applicant; the title of the invention; and the application filing date. One author (GC) charted the data.

### Data synthesis

Excel spreadsheets were used to chart the information from each source, and to organise the descriptive information. A narrative synthesis of the important underpinning concepts and challenging issues of digital twin science was based on an iterative scoping framework^15^ in which mapping key concepts and breadth of available information is emphasised over the depth of information from any one study.

## Results

### Type and recency of digital twin research

**Fig. 1:**
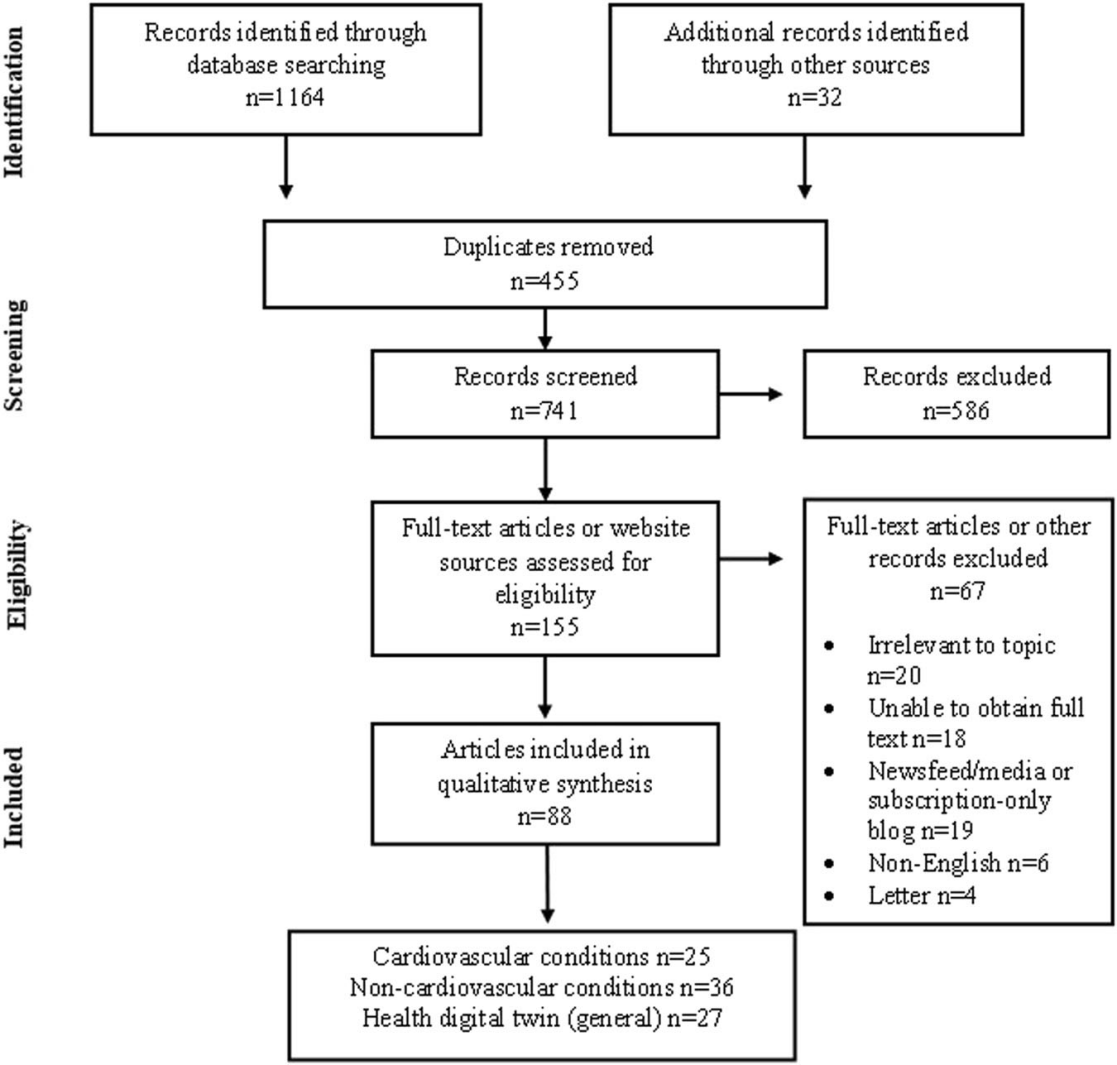
Flowchart of the search process.

The search of five electronic databases yielded 88 papers from 83 authors. (Fig. 1) Papers were original research (48%); reviews (24%); narratives (8%); book chapters (7%); conference papers (2%); commentary or viewpoint (10%) and position papers (1%). Overall, the papers related to cardiovascular conditions (28%, *n* = 25), non-cardiovascular conditions (41%, *n* = 36), and general aspects of the health digital twin (31%, *n* = 27). (Supplementary Table 1) CVD was the subspecialty with the majority of papers (*n* = 25); subspecialties within the 36 non-CVD papers included diabetes (*n* = 3), critical care (*n* = 4), cancer (*n* = 5), hepatology (*n* = 3), and multiple sclerosis (*n* = 2). Most of the papers were published between 2016 and 2021, underscoring the recency of health-related digital twin research. Geographically, the first author locations were worldwide: Europe (*n* = 35), North America (*n* = 15), UK (*n* = 13), India (*n* = 6), China (*n* = 5), Russia (*n* = 4), Australia (*n* = 3), Turkey (*n* = 1), and Morocco (*n* = 1). First author affiliations were universities and research institutes (*n* = 67), hospital centres (*n* = 4), private facilities or companies (*n* = 10), or a combination of these settings. The breadth of interdisciplinary sciences that converge in the field was evident in the predominance of the non-clinical sciences, such as bioengineering, robotics and cybernetics, mathematics, biophysics, mechanics and high-performance computing, suggesting their crucial influence in driving health digital twin research.

### Health digital twin: key concepts

**Fig. 2:**
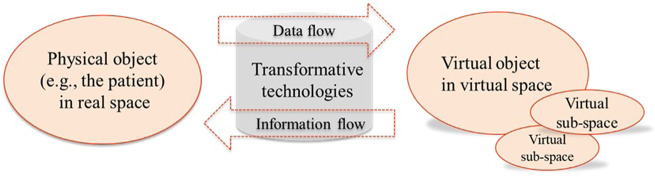
Concept of a cyber-physical-system, enabled by the convergence and synchronisation of physical and virtual systems^8^.

Regardless of the disease or condition in which digital twin research is applied, there are several shared, essential concepts (Box 1). First, the defining field of the computer sciences is artificial intelligence (AI). AI systems exploit processes that emulate human reasoning by using advances in four key fields, namely computational power, ‘big data’ processing, machine learning, and pattern recognition^16,17^. Second, the Internet of Things (IoT) refers to facilitating data exchange (including so-called ‘big data’) between different physical sources in a network^8,18^. IoT-enabled techniques for AI systems, combined with cloud computing, facilitate the creation of a digital profile of a real-world physical system^8^. Third, a digital twin requires bidirectional data exchange between the digital and physical twins on a continuous or at least periodic basis, which creates the characteristic cyber-physical system (CPS)^8^ (Fig. 2). The frequency of the data feed determines the relative passivity or activity of twin sub-types^19^. Fourth, whilst a digital twin can simulate a ‘what if’ scenario, the cumulative, real-time, real-world data exchange within the CPS gives the twin the further capacity for monitoring, diagnostics, and forecasting. This is enabled by what is known as closed-loop optimisation, whereby the constant synchronisation between the twins allows the virtual twin to quickly reconfigure as it adopts the properties of its physical twin, predicts problems, and tests potential solutions before deployment^8^. These more operational, intelligent elements differentiate a digital twin from a simulation-only model^8,20^.

The above capabilities—AI systems, IoT techniques and bidirectional data exchange—combine to surpass those of traditional data processing and improve the clinical utility of data-driven predictive and prognostic AI models^21^. For example, traditional data processing is typically associative, using epidemiological and statistical models based on structured, descriptive, retrospective data about a known population^17,21^. Such a data pool is more static than dynamic, usually accumulating steadily into local, centralised storage. Furthermore, data are often fragmented and isolated rather than merged with data from different sources, such as health records, wearable devices, and digital models^8,18^. In contrast, within the digital twin paradigm is the processing of high-dimensional, unstructured, decentralised data that accumulate prospectively and exponentially from multiple sources^17,21^, and contain (near) constant data flow from the environment, known as context-awareness^7^. Using a technique known as data fusion^12^, prescriptive- and predictive-type analyses of big data encompass so-called supervised and unsupervised machine-learning processes characterised, for example, by exploratory pattern recognition and use of physiologic, clinical, and social causal pathways in disease^21^. A subset of machine learning, known as deep learning and convolutional neural networks (Box 1), is posited to fundamentally change disease outcome prediction^17,22^. Taken together, the reasoning capability achievable with these data-driven technologies goes beyond both established processing with statistical techniques (such as logistic regression and decision trees^17^), and numerical, physics-based analyses as are used in haemodynamic models of coronary vessels^23^ or heart valve mechanics^24^.

#### Digital twin applications in cardiovascular disease

**Fig. 3:**
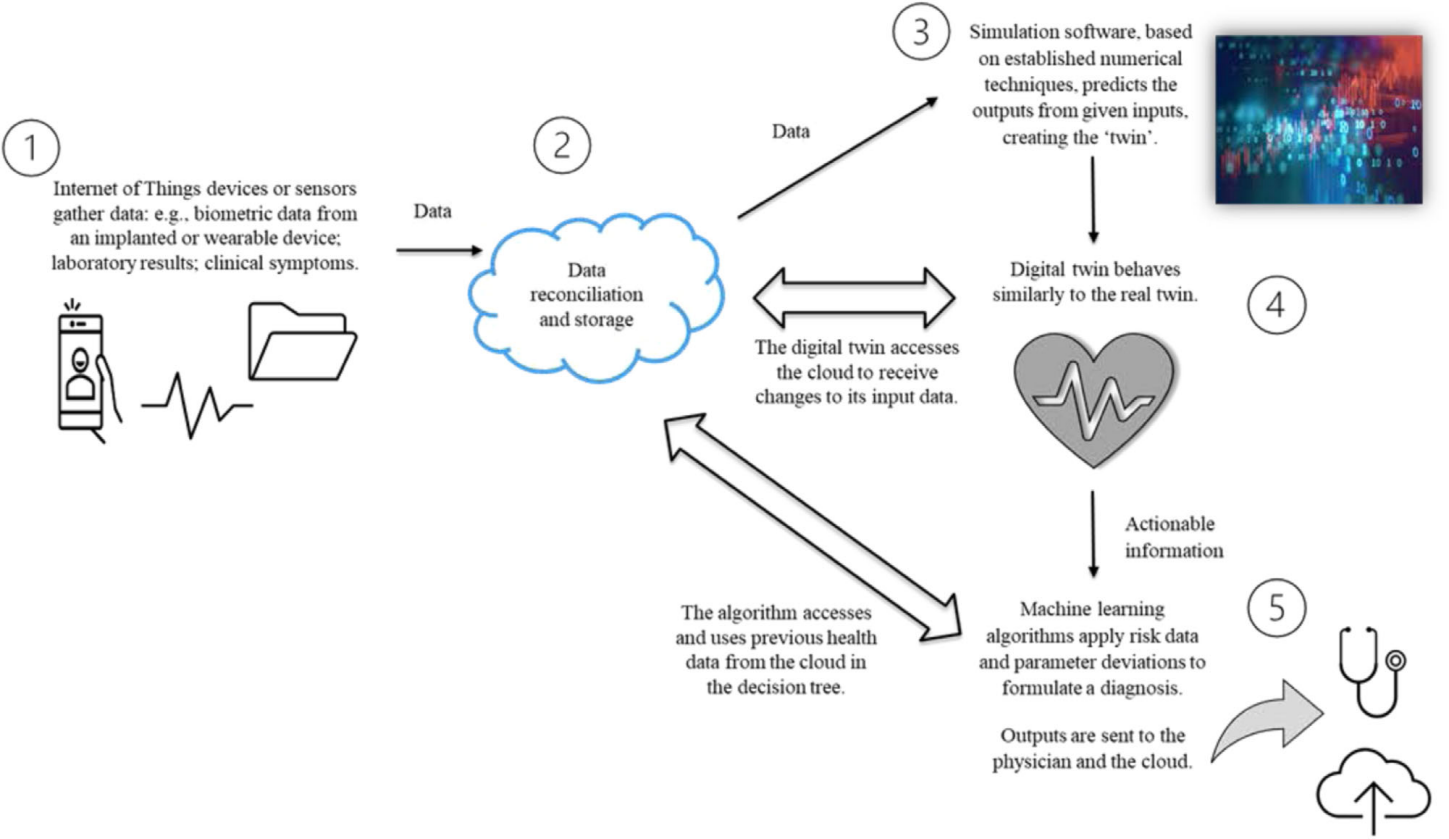
Concepts in a digital twin model of the heart^50^.

Research that used the term digital twin to describe models for addressing specific CVD-related clinical problems was detailed further (Table 1). Common to the methods used were simulation and modelling techniques from computing, bioengineering and mathematical sciences. The original research papers were best described as proof of concept and model validation; none were fully integrated into routine clinical practice, although seven were described as precursors to either an active or semi-active digital twin (Fig. 3). Nine discussion-type papers broadly examined precision cardiology in terms of machine-learning-based applications, combined statistical and mechanistic modelling techniques, clinical acceptability, translation, potential benefits, and limitations. In terms of scale, the digital twin elements were mostly applied at the organ level, modelled using one or more structural, biomechanical, and electrical characteristics of the heart derived from echocardiographic, tomographic, magnetic resonance and other imaging; also, electrocardiogram (ECG) databases and mathematical models. Two twin models using ‘human’ characteristics at the scale of a virtual patient incorporated descriptive data from health records and prospective biometric and behavioural data from smart wearables or other devices. One of the studies^25^ created a databank of profiles from existing electronic health records to derive multiple demographic and clinical variables against which to test antihypertensive treatment selection for patients. Using edge computing innovations in the context of ischaemic heart disease, another study^26^ used both an ECG database to train and test the AI dataset and smartphones paired with other external sensor devices, known as a body area network. Bluetooth connectivity and 5G network services communicated biometric data from the real twin’s smartphone to the digital twin in which the data fusion and analyses occurred.

**Table 1:** Digital twin-related applications in cardiovascular diseases.

Author(s)/year/first author country Target issue or overall aim	Research concept Modelling methods or twinning elements used	Status
**1. Endovascular repair**		
Auricchio et al. 2013 Italy^51^ AAA repair technique in a poor candidate for open surgery.	Compared pre-operative patient-specific simulation of the implant of a custom-made endograft prediction with post-operative outcomes. *Numerical analyses; FEA*	Proof of concept; **Precursor of a DT**
Biancolini et al. 2020 Italy^52^ High-fidelity surgical planning tool for thoracic aortic aneurysm repair to visualise, interactively and almost in real-time, the effect of various bulge shape parameters.	ROM framework to overcome the computing costs required in CFD techniques that are needed for blood flow prediction. *ROM, RBF, CFD*	Proof of concept; **Precursor of a DT**
Chakshu et al. 2020 UK^19^ Detection of AAA and severity classification using a virtual patient database.	Applies an inverse analysis system to blood flow prediction; and recurrent neural networks to classify AAA severity. *Deep learning and neural networks, waveform calculation/vessel dynamics, inverse analysis*	Model validation; **Precursor concept for an active DT**
Hemmler et al. 2019 Germany^53^ A DT for pre-operative selection of stent-graft size and material to overcome late complications of infrarenal endovascular repair versus open-surgical AAA repair.	Use of patient-specific pre-operative data and a morphing algorithm to predict post-operative graft configuration and wall stress; mechanical modelling of the graft and the geometry of aneurysms. *CFD, FEA*	Model validation
Larrabide et al. 2012 Spain^54^ To improve selection, safety, and accuracy of intracranial stent implantation for intracranial aneurysm using a novel virtual stent deployment.	Use of a ‘phantom’ and a digital replica to compare in vitro experiments with computational analysis of stent configurations within patient-specific anatomy and aneurysm geometry. *CFD, deformation models*	Computational model
**2.** **Ischaemic or occlusive disease and hypertension**		
Martinez-Velazquez et al. 2019 Canada^26^ Use of ‘edge computing’ means, e.g., body sensors, Bluetooth, and 5G networks, to detect and aggregate bio-signals into a DT interface for detecting dysrhythmias caused by a myocardial infarction.	Multilayer platform proposed in which a pipeline of AI-based analyses of ECG and biodata from the real twin (the IHD patient) in real-time builds a DT rendering of the heart. PTB Diagnostic ECG Database^55^ was used to train and test the CNN model. *Edge computing, AI, neural networks*	Proof of concept; **Precursor concept for an active DT**
Mazumder et al. 2019 India^27^ Training machine-learning algorithms with conventional mathematically-derived synthetic bulk data requires an alternative approach to improve the accuracy of simulated ‘what if’ scenarios for CAD with better pathophysiological interpretability.	The DT is modelled with a two-chambered heart and baroreflex-based blood pressure control to generate synthetic physiological data in healthy and atherosclerotic conditions. The MIMIC-II database^56^ was used to develop the PPG signal algorithm. *ROM of haemodynamics/ flow resistance; synthetic PPG signal data generation for training machine-learning algorithms*	Model validation
Naplekov et al. 2018 Russia^23^ A DT of coronary vessels can give a visual representation of the wearing process and progression of heart disease but requires haemodynamic and shear stress modelling.	Numerical simulation of the mechanical characteristics of the coronary vessel system, such as laminar and turbulent blood flow, and the impact of thrombus-induced vortex flow on load, blood pressure, and valves. *CFD*	Computational model
Semakova et al. 2018 Russia^25^ Data-driven DT profiles of real hypertensive patients can be used to facilitate virtual clinical trials that predict blood pressure variability and the effect of treatment.	Modelling of the annual average blood pressure variability and treatment effectiveness of antihypertensive drugs, based on diverse variables obtained from actual EMR data (*n* = 4521). *Probabilistic modelling/stochastic methods*	Clusters are precursors of a larger dynamic population model
Chakshu et al. 2019 UK^57^ Detection of carotid stenosis severity from a video of a human face.	In vivo head vibrations are compared against virtual vibration data generated from a coupled computational blood flow and head vibration model. *Principal component analysis*	Model validation; **Semi-active DT model**
Jones et al. 2021 UK^58^ Applies machine learning for the detection of stenoses and aneurysms, adopting algorithms that learn patterns and biomarkers from a labelled dataset.	Presents the ML methodology and metrics used for quantification of arterial disease classification accuracies using only pressure and flow-rate measurements at select locations in the arterial network. A freely available virtual patient database^59^ was used to train the algorithms. *ML methods: Naive Bayes, logistic regression, support vector machine, multilayer perceptron, random forests, and gradient boosting*	Proof of concept
Sharma et al. 2020 USA^60^ DT benefits are discussed in a hierarchy of AI applications in diagnostic and prognostic imaging, e.g., apparent superior diagnostic accuracy of coronary stenosis by machine-learning-based CT-FFR over CTA alone.	n/a	n/a
**3. Heart failure**		
Hirschvogel et al. 2019 Germany^61^ DT model to demonstrate a personalised model of the failing heart, vascular system, and BiVAD implant design.	Increasing ventricular augmentation is applied and the effect on patient-specific ventricular wall mechanics and geometry is modelled. *0-D and 3D geometry/echocardiography; deformation elastodynamics*	Proof of concept in vivo porcine model (*n* = 11)
**4. Electrophysiology**		
Pagani et al. 2021 Italy^62^ Reviews issues with integrating imaging, rhythm, and other clinical data into numerical models for patient-specific prediction in cardiac EP.	n/a	n/a
Gillette et al. 2021 Austria^63^ Generating high-fidelity cardiac digital twins comprises both anatomical (from tomographic data) and functional (inferred from ECG) twinning stages. This study addresses limitations for both stages that impede efficiency and accuracy for clinical utility.	Describes and demonstrates methodologies (parameter vector and fast-forward ECG model), to improve the value of a biophysically-detailed digital twin replicating ventricular EP. *Finite element analysis*	Proof of concept
Camps et al. 2021 UK^64^ Investigates new computational techniques for the efficient quantification of subject-specific ventricular activation properties using CMR-based modelling and simulation and non-invasive electrocardiographic data.	Describes a sequential Monte Carlo approximate Bayesian algorithm to conduct the simultaneous inference of endocardial and myocardial conduction speeds and the root nodes; quantified the accuracy of recovering these activation properties in a cohort of twenty virtual subjects. *Bayesian computation-based inference method*	Statistical method; **Precursor concept for a DT**
Gerach et al. 2021 Germany^65^ Bidirectional coupling or strong coupling is required to simulate physiological behaviour of the heart including mechano-electric feedback; adaptation of this framework allows personalisation from ion channels to the organ level enabling digital twin modelling.	Provides parameterisations of a fully coupled multi-scale model of the human heart, including electrophysiology, mechanics, and a closed-loop model of circulation; demonstrates model validity using a simulation on personalised heart geometry created from MRI data of a healthy volunteer. *Mathematical framework for geometry and deformation*	Model validation; **Precursor concept for a DT**
**5. Precision cardiology (general)**		
Bende et al. 2020 India^50^ Machine-learning algorithms can be trained using data from implanted devices, e.g., pacemakers, to create an updateable virtual organ using simulation software.	Demonstrates the simulation method to create a DT of the heart and tests the accuracy of the decision tree obtained for classifying disease severity. *Finite element analysis; machine learning*	Statistical method
Lamata P. 2018 UK^40^ Challenges with the use of machine learning to reason from data within statistical models for CVD prediction.	n/a	n/a
Lamata P. 2020 UK^28^ Risks and benefits for the cardiac DT of mechanistic and statistical models; strategies to improve how the latter use patterns in big data for CVD prediction.	n/a	n/a
Niederer et al. 2019 UK^66^ Describes biophysical models in cardiology and prediction models for dysrhythmia and heart failure therapies; outlines translational barriers to personalisation and uptake into clinical decision-making.	n/a	n/a
Niederer et al. 2020 UK^37^ Describes patient-specific cardiac models and how virtual patient cohort models are developed and validated, and how model uncertainty is quantified; also, potential and future applications of virtual cohorts.	n/a	n/a
Hose et al. 2019 UK^42^ Processes for cardiovascular models for clinical decision support and uptake of DT-related disciplines and sciences, such as AI.	n/a	n/a
Corral-Acero et al. 2020 UK^13^ Discussion of DT concepts and applications in precision cardiovascular medicine.	n/a	n/a
Peirlinck et al. 2021 USA^67^ Historical development of cardiac modelling; future roles; challenges for precision medicine.	n/a	n/a

*AAA* abdominal aortic aneurysm, *AF* atrial fibrillation, *AI* artificial intelligence, *BiVAD* biventricular assist device, *CAD* coronary artery disease, *CFD* computational fluid dynamics, *CHF* congestive heart failure, *CMR* cardiac magnetic resonance, *CNN* convolutional neural network, *CT* computed tomography, *CTA* computed tomographic angiography, *CT-FFR* computed tomography-fractional flow reserve, *DT* digital twin, *ECG* electrocardiogram, *EMR* electronic medical record, *EP* electrophysiology, *FEA* finite element analysis, *IHD* ischaemic heart disease, *MIMIC-II* Multiparameter Intelligent Monitoring in Intensive Care II, *MRI* magnetic resonance imaging, *PPG* photoplethysmogram, *RBF* radial basis functions, *ROM* reduced-order model.

Box 1 Important terms used in digital twin science

**Table:** 

AI, artificial intelligence	Computer science systems that perform human-like cognitive tasks^21^.
Big data	Data of large volume, high dimensionality, high heterogeneity, rapid acquisition and high value^8^.
Boundary conditions	Constraints applied in differential equations to close a system, such as mass flow at the inlet and pressure at the outlet; component of numerical modelling and CFD^42^.
CFD, computational fluid dynamics	Modelling in which numerical methods simulate fluid flow; used in biophysics to simulate blood flow and anatomically accurate vascular geometry^54^.
CNN, convolutional neural networks	Feature of deep learning (AI) in which inputs from large databases pass through multiple layers of algorithms, increasing the complexity of outputs from layer to layer^21^.
CPS, cyber-physical system	A set of physical entities (e.g., devices, equipment, humans) that interact with a virtual cyberspace through a communication network, culminating in the digital twin^8^.
Data fusion	Technique to integrate massive volumes of data from multiple sources; comprises data pre-processing, data mining, and data integration^12^.
Digital twin	Virtual representation of a physical individual that dynamically reflects molecular status, physiology, and lifestyle^33^. Twins may be active (receiving real-time data); passive (data are used to create an off-line model); or semi-active (a passive twin with some animations and dynamic components)^19^.
Edge computing	Computations occurring close to the data source device (e.g., a wearable or other IoT device); lowers bandwidth demand^26^.
FEA, finite element analysis	Numerical method to solve equations governing fluid flow or structural behaviour; used to create digital instances of human organs^50^.
In silico models	Simulation of cells or systems using mathematics and computers to construct virtual environments in which to test hypotheses^21^.
IoT, Internet of things	System of interrelated internet-enabled devices that transfer and converge data over network ecosystems without requiring human-to computer interaction^18^.
Machine learning	AI technique whereby computers construct algorithms from data to learn^16^.
ROM, reduced-order model	Technique to lower the dimensionality of a complex system; reduces computing costs of high-fidelity simulations^52^.

### CVD digital twin: industry participants

Recognising the expanding number of companies with a commercial interest in this fast-moving field, Supplementary Table 2 contains a listing that is illustrative of some of the available health digital twin or simulation modelling products for managing cardiovascular conditions, such as IHD, heart failure, and aneurysm repair. These products are mostly two- and three-dimensional computational models and software to assist device placement and haemodynamic modelling, rather than the definitive continuous bidirectional data exchange system of the physical and digital twin pair. The patent search identified 18 applications from 11 applicants, of which 73% were companies and 27% were universities. Three applicants had cardiac-related inventions and one of these was an author of an included paper^27^.

### Health digital twin feasibility and implementation

**Fig. 4:**
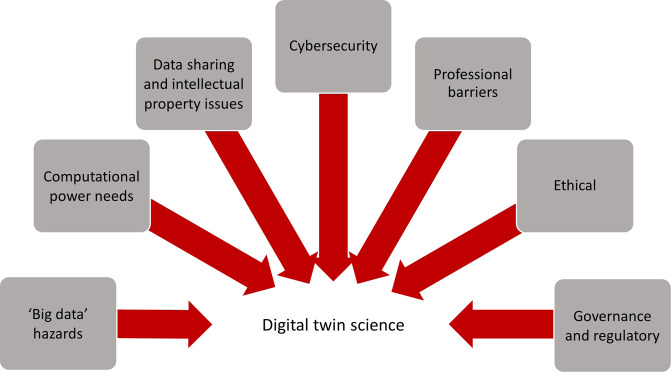
Translation issues in digital twin science.

Within the included papers, important shared translation issues for health digital twin research were illustrated by seven related themes (Fig. 4).

#### Big data hazards

Methodological hazards were a noted challenge to using AI for inductive reasoning; for example, the generalisability of findings necessitates external validation with new patient cohorts, or cohorts from different centres or different geographical locations, and across time^28^. Other risks could be confounding biases, perhaps whereby a variable in the vast data suggests a spurious association. Selection biases could affect conclusions or result in models that exacerbate racial or other societal biases and could be overcome by weighting, for example. Overall, big data compels transparency, the plausibility of computer-generated predictions, and external validation^28^.

#### Computational power needs

The Internet of Things as applied to healthcare presents challenges for devices with processor, memory and energy limitations. Computational power and the costs of related infrastructure that supports the acquisition, storage and processing of data are fundamental for big data sets. Scalability requirements for compatibility with shared networks are compounded by cybersecurity risks^29^.

#### Data sharing and intellectual property issues

Databank development and data sharing within and between countries compels compliance with data protection requirements around consent, anonymizing data, data breach notifications and safe international transfer. National and international data privacy laws safeguard individual health information but demand vigilance against both inadvertent and malicious breaches. The outputs of big data processing and machine learning raise considerations for personal, institutional and commercial intellectual property^30^.

#### Cybersecurity

Transformational technologies present demands on a medical CPS or digital twin software for confidentiality, reliability, safety, and secure coding, with minimal requirements for patching. Cloud computing systems are, like more conventional physical media, subject to vulnerabilities in computational, storage, and infrastructure resources. Third-party-related risks include eavesdropping, malware, and costly denial-of-service attacks^29^. As long as commercial entities have an interest in personal and medical data collection, storage, and analysis, research that requires data harvesting attracts understandable public aversity to big data use, misuse, breaches and theft that must be mitigated^31^.

#### Professional barriers

The credibility of virtual patient models to predict disease risk and progression in a real patient, and the trust required of the computational processes that deliver these, presents a potential barrier to their uptake into a routine workflow. Further, despite the centrality of patient care to the role of a physician, fear of replacement or de-skilling of the clinician specialist with software and other technology has been mooted as an obstruction to translation^32^ to be balanced against the promise of greater efficiency and individualisation^16^. Alongside the technology-related reservations are concerns that AI applications might jeopardise the important social interactions between colleagues and between clinicians and patients, affecting the central experience of both groups in medicine^32^.

#### Ethical barriers

Creating a digital twin of a patient for precision medicine raises considerable ethical questions around its legacy, privacy, and identity; and its termination when the real twin dies. Practices around informed consent must adapt to allow access to the electronic medical records of a large population, for example, to develop a databank representative of that population and to release it for research purposes. Questions of equity concern who has access to the benefits derived from people’s biological data. Unknown is whether health digital twin creation exacerbates racial or other societal biases or discriminates against the least well off in society, or against populations under-represented in model cohorts that lack demographic and ethnic heterogeneity^13^. Who misses out? Individuals already differ in strength, health, and longevity—if these differences are quantified in a person’s digital twin, and made available to the entire community will new issues around discrimination or equality emerge^33,34^?

#### Governance and regulatory

All potential regulatory and legal issues for a health digital twin are as yet uncertain but are likely to be especially demanding for approval of devices associated with medical cyber-physical systems that contain large amounts of embedded software for sensing and monitoring people’s activities. The complexity and interconnectedness of such devices may drive changes to how verification, scalability and evidence are documented and submitted. The sophistication of databases that need to be collated, curated and expanded to enable an active digital twin for routine clinical decision-making may limit submissions to regulators to more passive-type models that use twinning concepts. Although the approval process of regulatory bodies may differ in purpose, cost, timeline, and perceived rigour between countries or regions^35^, products that use personalised computational modelling and simulation for procedure planning purposes are currently available in the market (see Supplementary Table 2 for examples of products marketed for use in structural heart disease, cardiac catheterisation, and aneurysm repair).

As this science becomes more visible in the marketplace the requirements for certification and approval may drive changes to the traditional processes used by national regulatory agencies. For example, in aiming to accelerate and streamline the product development process for device manufacturers, the United States Food and Drug Administration recently commenced a programme of pre-qualifying appropriate evaluation tools (such as a digital tool or a computer model) for later use in the actual regulatory submission stage (https://www.fda.gov/medical-devices/science-and-research-medical-devices/medical-device-development-tools-mddt). This aligns with recognition within its strategic priorities of both the growing area of simulation software as a medical device and the potential for computational modelling and AI tools to aid device evaluation and reduce costs in the overall regulatory pipeline^36^.

Governance mechanisms needed to safeguard the rights of persons with a digital twin could, for example, draw from existing practices for how medical databases and biobanks are designed, regulated, and inspected. Existing privacy safeguards may require strengthening when genomics data are expanded with biological and behavioural data^29,33^. Therefore, relevant legislation—timely and harmonised across jurisdictions—must develop and adapt in step with the adjustments made by other agencies responding to the repercussions of technological change in this field. Collaborations between academia, industry and government may benefit the standardisation of methods and interoperability of software and other protocols.

## Discussion

This review explored the use of the term digital twin and its core concepts, chief of which is the notion of a continuous real-time multi-source data feed into the virtual twin, and the AI technologies that optimise these data into useful information about the physical twin. As intelligent systems that efficiently characterise, understand, cluster and classify complex data, health digital twins are proposed to augment, rather than displace, human intelligence in diagnostic and prognostic decisions in disease^8^. Estimating and stratifying risks, forecasting progression, choosing an intervention, and predicting its outcome using data streamed and integrated from many sources capture the role for digital twins in realising the possibilities of precision medicine. Importantly, the interplay of inductive and deductive reasoning underpins these operations within the cardiovascular digital twin model.

For CVD management, the potential for AI systems to more accurately phenotype patients with the same presentation or condition and overcome limitations of current risk-stratification algorithms could enable therapy selection that is based less on the responses of an average person than on the responses predicted in an individualised model^33^. Predictions about the best treatment for an individual would shift from being based on their current or past condition to being evaluated in the light of a future-facing simulation^37^. However, being able to collect and integrate in real time the multiple changeable molecular, physiological, behavioural, and other attributes of an actual person into a twin, then extract precise data for an intelligent digital representation is complex, and many obstacles to translation must be resolved. Among these challenges are computational power needs, cybersecurity concerns, data sharing issues, myriad ethical constraints, and barriers to adoption by clinicians of disease management decision tools derived from artificial intelligence systems.

Importantly, the characterisation of an active health digital twin as elucidated in this review is recent, relative to established complex modelling science used to make treatment predictions and inferences in disease, including CVD^38,39^. Use of the term digital twin in mechanistic models may be restrained by the absence of the definitive bidirectional data flow with a real patient, due to the extreme complexity required of a model to realistically be able to make such a claim. For example, the iterative process by which imaging data and engineering sciences combine towards an archetypal digital twin for clinical translation (figure in Box 2) underscores that clean separation between numerical modelling applications and the data-driven twin concept as identified in this review may be unrealistic. More compelling is the suggestion that synergistic mathematical (deductive) and data-driven (inductive) modelling could overcome limitations within, and build the links between, the information derived from each approach^13,40^. For example, within the digital twin construct and validation centred on a population-based databank, complementary deductive and inductive data modelling processes are at play^13^. The deductive, mechanistic model integrates clinical and experimental data to identify mechanisms and predict outcomes based on anatomical and mechanical knowledge of a physical system and hypothesised relationships^13^. Furthermore, such extensive system knowledge presents a descriptive advantage over an inductive or empirical model, and avoids the need to re-train a predictive mechanistic model for unseen data or new situations^41^. The advantages for clinical interpretability of such simulations may be constrained by the assumptions that are applied^13^, including the choice and impact of boundary conditions (Box 1) for measurements required to solve equations^42^. In contrast, the inductive, statistical pathway trains, tests, and revises complex data using machine-learning processes. It finds predictive relations, patterns and correlations when mechanisms are poorly understood, too complex to model mechanistically, or when missing data must be inferred^13^. An advantage of such empirically derived models is the capacity to efficiently process numerous multivariable data from, for example, biologic databases, wearable sensors, and other external sources^8^. Constraints may relate to the available volume and heterogeneity of data variables with which to train machine-learning systems^13^, and lower generalisation compared with mechanistic or physics-based models^43^. Furthering digital twin science will therefore likely continue to harness both data-driven and knowledge-based mathematical modelling. At the scale of the heart, for example, the feasibility of inductive-deductive model synergy for predicting conduction abnormalities has been reported from a retrospective study of aortic valve recipients^44^. In particular, use of machine learning to classify anatomical, procedural and mechanistic data augmented more traditional device-anatomy simulations to expand and integrate the available variables for reliably predicting new onset of bundle branch block or permanent pacemaker placement^44^. Acknowledgement of the achievements to date and substantial future challenges to integrate multiple physiological body systems is described in the widely-cited international initiative, the Virtual Physiological Human (https://www.vph-institute.org/projects.html).

This review concurs with other observations that digital twin research applications comprise elements, rather than all components, of the archetypal synchronised cyber-physical system supported by IoT connectivity^13^. Consequently, comparison of research and implementation would be aided by greater consensus about the digital twin definition^45^. Another important priority is to focus research into intuitive accessible ways for non-technical end-users to interact with digital twin systems in their field^45^. Future innovations include refining the visualisation methods by which non-expert users of a mirrored, networked system interact with the AI-derived information about the physical twin^45^. Augmented reality^45,46^, multidimensional holographic projections^10,29^, and 3D avatars^8^ are available methods; however, standardisation between multiple potential digital twins in a system would facilitate both their seamless interaction in the network connecting the virtual and physical twins, and the participation of human non-experts of data science^45^. The industry sector is expected to drive the key advances in the technologies that enable digital twin systems, for example, big data analytics platforms that favour localised over centralised storage, and parallel over serial processing of structured and unstructured data^43^. Improving IoT technologies will include developments in communication infrastructure such as wider availability of 5G mobile and internet, lower-cost sensor devices and flexible cloud and edge computing environments to meet the storage and processing needs of these networked services^43^. Further challenges are to ensure the timely and accurate update and replication of data derived from complex components of a physical twin (such as a human organ) into the digital twin to avoid delaying critical intervention in the physical twin^29^. Multiple digital twins within a shared interaction (such as the human body) need to exchange and synchronise information, requiring advancements in application programming interfaces that optimise consistent and predictable software-to-software interoperability^29,45^. Important developments within physics-based modelling involve improved affordability and fidelity of computational hardware to enable the equations governing a physical system (e.g., how vessels deform or interact with turbulent blood flow) to be solved faster; to improve the quality of 3D visualisations; and to accelerate the availability of training data sets for machine-learning models that are needed to create the digital twin^43^.

In the evolution of model-based personalisation for decision support, at least two socio-technical considerations of how such innovations flow into practice are essential for the clinical end-user: first, the accuracy, reproducibility, and consistency of data; and second, that measurement uncertainty is introduced into the data assimilation within the model^42^. Model-based applications lacking details of the effects of uncertainties within the clinical data that underpin the model can make the so-called black-box nature of AI feel uncomfortable and unrelatable to clinicians^42^. Therefore, among the challenges surrounding adoption is to optimise transparency around the evidence supporting the development and validation of a digital twin solution to aid treatment decisions or prognostic conclusions^47^. The progressive integration of AI-enabled platforms into traditional approaches to patient care will likely require the collaborative expertise from technological, biomedical, and behavioural sciences to cultivate conditions for uptake and routine use^48^. As models are validated in terms of an initial concept, a crucial step is extending the model to a more general patient cohort, with less controlled physiological and demographic characteristics^13^. The challenge of accessing large populations and their detailed information requires the development of so-called mega cohorts—prospective digital health ‘data lakes’; and big data infrastructure (including the techniques characterised as data fusion [Box 1]) to phenotype volunteers enrolled in these initiatives and to optimise the use, re-use, and sharing of these data^13,49^. Several established databanks supply anonymised data to researchers worldwide (National Institutes of Health, www.allofus.nih.gov/; UK Biobank, www.ukbiobank.ac.uk).

Among the strengths of this review is that its mapping intent enabled use of internet-sourced material alongside at least six types of scholarly publications, thus giving a fuller picture of the variety of disease conditions where digital twin science is being applied, and of the academic and non-academic stakeholders within this multidisciplinary field. Within an exploratory review of the term digital twin, several limitations should be noted. In taking a pragmatic approach to isolate work termed a digital twin, the search process may have excluded relevant papers describing predictive models but without explicit use of the term. Thus, the extent of digital twins for CVD in the published literature may be understated. We used a systematic search strategy within a mapping style of review; future studies of the field could alternatively conduct a systematic review of a narrower question and a smaller pool of papers of more uniform study design. The methodology assessments within that type of review would produce other perspectives on the topic. Material obtained from electronic databases and websites represents information available on the search date, risking that the review under-represents all available information on a rapidly advancing subject. The use of only English language sources potentially under-recognises all relevant literature in the field. One author charted the information from the various sources, risking that screening decisions could have resulted differently with multiple reviewers. Further, the potential has been mooted for AI and digital twin technology to gather ‘intelligence’ from vast volumes of patient data for optimising the patient experience, care coordination, scheduling, and other service-oriented operations at healthcare facility level^7,8^, but was not reviewed. The included papers did not evaluate any hypothesised economic benefits of digital twins applied to CVD management and this is an important area of future study.

In conclusion, digital twin research for CVD overall encompasses proof-of-concept studies illustrating the use of the data-driven approaches that typify the goals of precision medicine. The promise of an active digital twin of either the human heart or the human organism for clinical decision-making remains futuristic. Its advancement holds feasibility and implementation challenges that are not unique to CVD, namely that although grounded in AI it hinges on many human factors: citizen populations willing to prospectively contribute biodata towards mega cohorts of patients; an adaptive, agile ethical and regulatory landscape; and multidisciplinary scientific expertise. Review of the health digital twin term, concepts, and uses offered important contextual insights to the field in general and for CVD in particular for the recent collaborative workshop of clinicians, (bio)engineers, data scientists, and others required to advance research pathways for CVD, hosted by the University of Sydney Cardiovascular Initiative.

Box 2 Steps towards the construction of a digital twin for right ventricle to pulmonary artery conduits using in silico design and computational analysis of flow Pre-operative scans can now be used to reconstruct the patient’s anatomy in three dimensions. This model can be 3D printed to allow the surgeon to compare different strategies for virtual surgery. Modern 3D graphical rendering means that the model could be projected in space and the surgeon could “step inside the heart and move around”, facilitating discussion among clinicians. It is a short step to allow virtual surgery to be performed and the geometry modified.

In parallel, computational models are being used to study flow in the heart and through valves and stents. Currently, these models are too computationally demanding to run in real time. However, simulations of the pre-operation flow can be made and then the geometry can be modified to simulate a post-operative state. This is the start of the digital twin processes. Next, simulations for different levels of exercise and therefore blood flow rates can be made and used to develop a reduced-order model, the engineering core of a digital twin.

**Figure:**
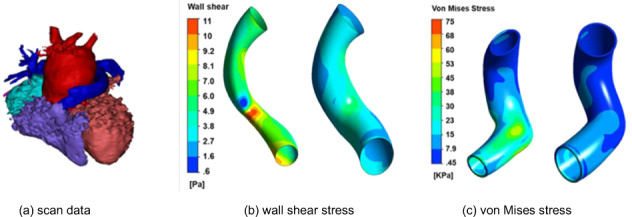
**Process of right ventricle to pulmonary artery conduit design.** **a** imaging of the heart to extract the shape of the right ventricle conduit; **b**, **c** results of simulations to determine the wall shear stress and the stress in the conduit wall. The left-side image is for the existing conduit and the right-side image is for a modified shape that improves the conduit performance. This article was published in JTCVS Open, 1, Ebrahimi, P. et al. Evaluation of personalised right ventricle to pulmonary artery conduits using in silico design and computational analysis of flow. 33–48, Copyright Elsevier (2020).

### Reporting summary

Further information on research design is available in the Nature Research Reporting Summary linked to this article.

## Supplementary information

Supplementary Information

Reporting Summary

## Data Availability

The datasets supporting the conclusions of this article are included within the article and its additional files.
