# Supplementary material for: The health digital twin to tackle cardiovascular disease—a review of an emerging interdisciplinary field
Source: NPJ Digit Med. 2022 Aug 26;5:126. doi: 10.1038/s41746-022-00640-7 (PMC9418270; doi:10.1038/s41746-022-00640-7)
Supplement: Supplementary file 1 — Supplementary Information [file 41746_2022_640_MOESM1_ESM.pdf]

Supplementary Note 1

EMBASE Search

1 ("digital twin*" or "digital replica*").mp. [mp=title, abstract, heading word, drug trade name, original title, device manufacturer, drug manufacturer, device trade name, keyword, floating subheading word, candidate term word]
2 ("virtual patient*" or "virtual patient cohort*" or "precision medicine" or "personali?ed medicine" or medic* or health or disease or diagnos*).mp. [mp=title, abstract, heading word, drug trade name, original title, device manufacturer, drug manufacturer, device trade name, keyword, floating subheading word, candidate term word]
3 1 and 2
4 surg*.mp.
5 exp cardiovascular disease/ or cardiovascular disease*.mp.
6 cardi*.mp.
7 cardiology/ or cardiology.mp.
8 cancer*.mp. or exp Neoplasms/
9 Leuk?emia.mp. or exp leukemia/
10 malignan*.mp.
11 tumo?r*.mp.
12 pulm*.mp.
13 exp lung diseases/ or lung.mp. or lung*.mp. [mp=title, abstract, heading word, drug trade name, original title, device manufacturer, drug manufacturer, device trade name, keyword, floating subheading word, candidate term word]
14 hepat*.mp.
15 liver.mp. [mp=title, abstract, heading word, drug trade name, original title, device manufacturer, drug manufacturer, device trade name, keyword, floating subheading word, candidate term word]
16 (intensive care unit or ICU).mp. [mp=title, abstract, heading word, drug trade name, original title, device manufacturer, drug manufacturer, device trade name, keyword, floating subheading word, candidate term word]
17 intensive care.mp.
18 critical care.mp.
19 exp neurology/
20 exp nervous system disease/
21 neuro*.mp.
22 exp brain disease/ or brain.mp. or brain.mp. [mp=title, abstract, heading word, drug trade name, original title, device manufacturer, drug manufacturer, device trade name, keyword, floating subheading word, candidate term word]
23 diabetes.mp.
24 4 or 5 or 6 or 7 or 8 or 9 or 10 or 11 or 12 or 13 or 14 or 15 or 16 or 17 or 18 or 19 or 20 or 21 or 22 or 23
25 1 and 24

Patents Websites

Terms used were “digital twin” AND “heart”; “digital twin” AND “disease”; “digital twin” AND “vascular”; “digital twin” AND “vessel”; and “digital twin” AND “patient”.

Supplementary Table 1. Features of included papers
Superscripts refer to the reference list in the main manuscript.

Author(s)/first author country	Description	Condition or topic
**Part a: Cardiovascular conditions**		
**2012**		
Larrabide et al. Spain [54]	original research	intracranial aneurysm
**2013**		
Auricchio et al. Italy [51]	original research	AAA repair
**2018**		
Lamata P. UK [40]	commentary	precision cardiology
Naplekov et al. Russia [23]	original research	sheer stress in coronary vessels
Semakova et al. Russia [25]	original research	hypertension
**2019**		
Chakshu et al. UK [57]	original research	carotid stenoses
Hemmler et al. Germany [53]	original research	AAA repair
Hirschvogel et al. Germany [61]	original research	CHF
Hose et al. UK [42]	position paper	precision cardiology
Martinez-Velazquez et al. Canada [26]	original research	IHD
Mazumder et al. India [27]	original research	CAD
Niederer et al. UK [66]	review	computational heart models
**2020**		
Biancolini et al. Italy [52]	original research	thoracic aortic aneurysm repair

Bende et al. India [50]	original research	simulation method for a cardiac DT
Chakshu et al. UK [19]	original research	AAA repair
Corral-Acero et al. UK [13]	review	precision cardiology
Lamata P. UK [28]	review	precision cardiology
Niederer et al. UK [37]	review	virtual patient cohorts
Sharma et al. USA [60]	review	cardiothoracic imaging
**2021 (to 09 August)**		
Camps et al. UK [64]	original research	personalisation of EP properties in cardiac models
Gerach et al. Germany [65]	original research	mechanics, EP, and circulation in a multi-scale cardiac model
Gillette et al. Austria [63]	original research	digital twins of ventricular EP
Jones et al. UK [58]	original research	stenoses and aneurysms
Pagani et al. Italy [62]	review	integrating clinical and EP data into mathematical models
Peirlinck et al. USA [67]	review	precision cardiology

**Part b: Non-cardiovascular conditions**		
**2016**		
Golse et al. France<br>Predicting the risk of post-hepatectomy portal hypertension using a digital twin: A clinical proof of concept. J Hepatol, 2021. 74(3): p. 661-669.	original research	liver failure
**2019**		
Bjornsson et al. Sweden<br>Digital twins to personalize medicine. Genome Med, 2019. 12(1): p. 4.	original research	drug selection
Fisher et al. USA<br>Machine learning for comprehensive forecasting of Alzheimer's Disease progression. Scientific Reports, 2019. 9(1): p. 13622.	original research	Alzheimer's disease
Lauzeral et al. France<br>A model order reduction approach to create patient-specific mechanical models of human liver in computational medicine applications. Computer Methods and Programs in Biomedicine, 2019. 170: p. 95-106.	original research	liver surgery
Liu et al. China<br>A novel cloud-based framework for the elderly healthcare services using digital twin. IEEE Access, 2019. 7: p. 49088-49101.	review	aged care

Özdemir V. Canada<br>Genomics, the internet of things, artificial intelligence, and society, in Applied Genomics and Public Health, G.P. Patrinos, Editor. 2020, Academic Press. p. 275-285.	book chapter	genomics
Pizzolato et al. Australia<br>Neuromusculoskeletal modeling-based prostheses for recovery after spinal cord injury. Front Neurorobot, 2019. 13: p. 97.	review	spinal cord injury
Vasilieva et al. Russia<br>The light and the dark sides of the moon: possibilities of Industry 4.0 for early non-invasive diagnosis of pathology and health-risks. J Physics: Conference Series, 2019. 1379: p. 012070.	commentary	psychiatry
**2020**		
Bertolini et al. USA<br>Modeling disease progression in mild cognitive impairment and alzheimer's disease with digital twins. 2020. arXiv:2012.13455.	original research	Alzheimer's disease
Defraeye et al. Switzerland<br>Predicting transdermal fentanyl delivery using mechanistic simulations for tailored therapy. Front Pharmacol, 2020. 11: p. 585393-585393.	original research	transdermal drug delivery

Di Filippo et al. Italy<br>Single-cell digital twins for cancer preclinical investigation, in Metabolic Flux Analysis in Eukaryotic Cells: Methods and Protocols, D. Nagrath, Editor. 2020, Springer US: New York, NY. p. 331-343.	original research	cancer
Farkas et al. Czech Republic<br>The role of the combined use of experimental and computational methods in revealing the differences between the micron-size particle deposition patterns in healthy and asthmatic subjects. J Aerosol Sci, 2020. 147: p. 105582.	original research	asthma
Fu et al. USA<br>iPhantom: a framework for automated creation of individualized computational phantoms and its application to CT organ dosimetry. 2020. arXiv:2008.08730.	original research	CT dosimetry
Geissler et al. Germany<br>Personalized computed tomography – Automated estimation of height and weight of a simulated digital twin using a 3D camera and artificial intelligence. Rofo, 2021. 193(04): p. 437-445.	original research	CT imaging
Gkouskou et al. Greece<br>The “virtual digital twins” concept in precision nutrition. Adv Nutrition, 2020. 11(6): p. 1405-1413.	review	obesity and nutrition

Gomis-Fons et al. Sweden<br>Model-based design and control of a small-scale integrated continuous end-to-end mAb platform. Biotech Progr, 2020. 36(4): p. e2995.	original research	laboratory science
Goodwin et al. Australia<br>A systematic stochastic design strategy achieving an optimal tradeoff between peak BGL and probability of hypoglycaemic events for individuals having type 1 diabetes mellitus. Biomedical Signal Processing and Control, 2020. 57: p. 101813.	original research	type 1 diabetes
Kumar et al. India<br>Adaptations of pharma 4.0 from industry 4.0. Drug Invention Today, 2020. 14(3).	review	pharmaceutics
Lal et al. USA<br>Development and verification of a digital twin patient model to predict specific treatment response during the first 24 hours of sepsis. Crit Care Explor, 2020. 2(11): p. e0249-e0249..	original research	sepsis
Lal et al. USA<br>Artificial intelligence and computer simulation models in critical illness. World J Crit Care Med, 2020. 9(2): p. 13-19	review	critical care
Malone et al. Germany<br>Artificial intelligence predicts the immunogenic landscape of SARS-CoV-2 leading to universal	original research	vaccines

blueprints for vaccine designs. Scientific Reports, 2020. 10(1): p. 22375.		
Petrova-Antonova et al. Bulgaria<br>A digital twin platform for diagnostics and rehabilitation of multiple sclerosis. in computational science and its applications – ICCSA 2020. 2020. Cham: Springer International Publishing.	book chapter	multiple sclerosis
Shamanna et al. India<br>Reducing HbA1c in type 2 diabetes using digital twin technology-enabled precision nutrition: a retrospective analysis. Diab Therapy, 2020. 11(11): p. 2703-2714.	original research	type 2 diabetes
Subramanian K. USA<br>Digital twin for drug discovery and development—the virtual liver. J Indian Inst Sci, 2020. 100(4): p. 653-662.	original research	liver injury
Walsh et al. USA<br>Generating digital twins with multiple sclerosis using probabilistic neural networks. 2020, Cold Spring Harbor Laboratory Press: Cold Spring Harbor.	original research	multiple sclerosis
Zhang et al. China<br>Cyber resilience in healthcare digital twin on lung cancer. IEEE Access, 2020. 8: p. 201900-201913.	original research	lung cancer

Zhou et al. China<br>Virtual patients for mechanical ventilation in the intensive care unit. Comp Methods Progr Biomed, 2021. 199: p. 105912.	original research	mechanical ventilation
**2021 (to 09 August)**		
Bethencourt et al. France<br>Guiding measurement protocols of connected medical devices using digital twins: A statistical methodology applied to detecting and monitoring lymphedema. IEEE Access, 2021. 9: p. 39444-39465.	original research	lymphoedema
Caligiore et al. Italy<br>Increasing serotonin to reduce parkinsonian tremor. Front Syst Neurosci, 2021. 15: p. 682990-682990.	original research	Parkinson's disease
Calka et al. France<br>Machine-Learning based model order reduction of a biomechanical model of the human tongue. Comp Methods Progr Biomed, 2021. 198: p. 105786.	original research	oral surgery
Dang et al. USA<br>Predictive modeling in neurocritical care using causal artificial intelligence. World J Crit Care Med, 2021. 10(4): p. 112-119.	review	critical care
Hernigou et al. France	original research	ankle surgery

Digital twins, artificial intelligence, and machine learning technology to identify a real personalized motion axis of the tibiotalar joint for robotics in total ankle arthroplasty. Int Orthop, 2021.		
Pizzolato et al. Australia<br>Non-invasive approaches to functional recovery after spinal cord injury: Therapeutic targets and multimodal device interventions. Exp Neurol, 2021. 339: p. 113612.	review	spinal cord injury
Shamanna et al. India<br>Retrospective study of glycemic variability, BMI, and blood pressure in diabetes patients in the digital twin precision treatment program. Scientific Reports, 2021. 11(1): p. 14892-14892.	original research	type 2 diabetes
Wan et al. China<br>Semi-supervised support vector machine for digital twins based brain image fusion. Front Neurosci, 2021. 15: p. 705323-705323.	original research	brain imaging
Walter et al. Germany<br>How artificial intelligence might disrupt diagnostics in hematology in the near future. Oncogene, 2021. 40(25): p. 4271-4280.	review	haematology oncology

**Part c: Health digital twin - general**		
**2017**		
Patterson et al. UK<br>A framework to establish credibility of computational models in biology. Prog Biophys Molec Biol, 2017. 129: p. 13-19	narrative	model validation framework
**2018**		
Bruynseels et al. Netherlands [33]	narrative	ethical implications
Verghese et al. USA [32]	viewpoint	AI and the physician role
**2019**		
Bagaria et al. Canada<br>Health 4.0: Digital Twins for Health and Well-Being, in Connected Health in Smart Cities, A. El Saddik, M.S. Hossain, and B. Kantarci, Editors. 2020, Springer International Publishing: Cham. p. 143-152.	book chapter	concepts and applications
Barricelli et al. Italy [8]	review	concepts and applications
Kendzierskyj et al. UK [31]	book chapter	concepts, data security, governance

**2020**		
Ahmadi-Assalemi et al. UK [18]	book chapter	concepts, definitions, frameworks, data security,
Bhattad et al. USA [16]	review	concepts
Croatti et al. Italy [10]	narrative	concepts and applications
de Boer B. Netherlands [34]	narrative	concepts and the phenomenology of medicine
Erol et al. Turkey [46]	narrative	DT technology for multisector uses
Erol et al. Turkey [9]	narrative	healthcare applications; commercial products
Fagherazzi G. Luxembourg [49]	viewpoint	digital and biological phenotyping
Georges-Filteau et al. Netherlands Synthetic Observational Health Data with GANs: from slow adoption to a boom in medical research	review	synthetic data concept and uses

and ultimately digital twins? arXiv preprint arXiv:2005.13510, 2020.		
Ghita et al. Morocco Digitalization against the new outbreak. in 2020 Fourth World Conference on Smart Trends in Systems, Security and Sustainability (WorldS4). 2020.	conference paper	civic pandemic management
Jimenez et al. UK [29]	book chapter	DT technology concepts, definitions
Lhotska L. Czech Republic Application of industry 4.0 concept to health care. Stud Health Technol Inform, 2020. 273: p. 23-37.	narrative	industry 4.0 concepts in health care
Mohapatra et al. India An appraisal of literature for design and implementation of developing a framework for digital twin and validation through case studies. Health and Technology, 2020. 10(5): p. 1229-1237.	review	DT validation using case studies
Rivera et al. Canada [30]	conference paper	concepts and technology systems
Sinisi et al. Italy Optimal personalised treatment computation through in silico clinical trials on patient digital	original research	computation method for DT creation and use

twins. Fundamenta Informaticae, 2020. 174(3-4): p. 283-310.		for in-silico clinical trials
**2021 (to 09 August)**		
Braun M. Germany<br>Represent me: please! Towards an ethics of digital twins in medicine. J Med Ethics, 2021. 47(6): p. 394.	commentary	ethical implications
Kamel Boulos et al. China<br>Digital twins: from personalised medicine to precision public health. J Personal Med, 2021. 11(8): p. 745.	review	DT concepts, potential, challenges
Krutzinna J. Norway<br>Simulating (some) individuals in a connected world. J Med Ethics, 2021. 47(6): p. 403.	commentary	ethical implications
Lupton D. Australia<br>Language matters: the ‘digital twin’ metaphor in health and medicine. J Med Ethics, 2021. 47(6): p. 409.	commentary	ethical implications
Mittelstadt B. UK<br>Near-term ethical challenges of digital twins. J Med Ethics, 2021. 47(6): p. 405.	commentary	ethical implications
Nyholm D. Netherlands	commentary	ethical implications

Should a medical digital twin be viewed as an extension of the patient's body? J Med Ethics, 2021. 47(6): p. 401-402.		
Volkov et al. Russia Digital twins, internet of things and mobile medicine: a review of current platforms to support smart healthcare. arXiv preprint arXiv:2106.11728, 2021.	review	DT technology platforms, definitions

Abbreviations: AAA, abdominal aortic aneurysm; AI, artificial intelligence; CAD, coronary artery disease; CHF, congestive heart failure; CT, computed tomography; DT, digital twin; EP, electrophysiology; IHD, ischaemic heart disease.

Supplementary Table 2. Examples of CVD-related digital twin or modelling products

Company	Product and target condition
ADAS 3D	Software that uses cardiac MRI data to visualise atrial or ventricular fibrosis before and during **ablation procedures**.<br>www.adas3d.com/en/adas-vt-technology.html
Ansys	Simulation and modelling of computational fluid dynamics, finite element analysis, and fluid-structure interaction of implantable cardiac devices **(stents, pacemakers, valves)**<br>www.ansys.com/solutions/solutions-by-industry/healthcare/cardiovascular
Corify	Acorys® is a non-invasive electrocardiographic imaging device aimed at improving precision in ablation procedures for **atrial fibrillation**.<br>www.corify.es/solution/
Dassault Systèmes	Living Heart Project: transforms a 2D into a 3D model with blood flow, mechanics and electrical impulses; available to researchers or clinicians to run a hypothetical scenario (e.g., adding **pacemakers and implantable devices**).<br>www.3ds.com/products-services/simulia/solutions/life-sciences/the-living-heart-project/
ELEM	Supplier of cloud-based virtual models of cardiac structures to the MedTech and pharmaceutical industries to facilitate product development by coding the models to simulate pathology conditions.<br>www.elem.bio/elem-technology.html

FEops	Cloud-based HEARTguide simulation software to create digital replicas and 3D views from a cardiac valve image to assist with planning surgical repair **(transcatheter aortic valve implantation)**. Availability for clinical or non-clinical use varies by location.<br>www.feops.com/
HeartFlow	Cloud-based service enabling clinicians to identify **coronary artery disease** based on computed tomography images of a patient's heart; uses a fluid dynamic model of the blood flow through the coronary vessels to identify significant stenoses without the need for invasive angiography.<br>www.heartflow.com/heartflow-ffrct-analysis/
inHeart	Cloud-based software for the analysis of cardiac CT and MRI data to generate 3D chamber anatomy maps to assist atrial and ventricular **ablation procedures**.<br>www.inheart.fr/
Medtronic	CardioInsight is an ECG mapping tool with two inputs: electrical data obtained from a wearable vest and CT-derived cardiac anatomical data. The software displays and analyses an ECG in 3D maps (**atrial and ventricular dysrhythmias)**.<br>www.medtronic.com/us-en/healthcare-professionals/therapies-procedures/cardiac-rhythm/cardioinsight-cardiac-mapping.html/
NTT Research	Project to develop and validate computational models, implemented on a digital twin platform representing heart and vascular dynamics **(myocardial infarction and acute heart failure**).<br>www.ntt-research.com/press-release/ntt-research-and-ncvc-to-collaborate-on-cardiovascular-models-and-bio-digital-twin-applications/

Numericor	Multiple software products for simulating cardiac electrophysiology, mechanics and hemodynamics at cellular, tissue and organ scales, including in-silico twin models. Targets MedTech and research users.<br>www.numericor.at/
OxfordHeartbeat	PreSize is simulation software to aide deployment of endovascular devices into an **intracranial aneurysm.**<br>www.oxfordheartbeat.com/technology/
Philips	HeartModel: an application that generates 3D views of the left heart chambers and pumping dynamics **(heart failure).**<br>HeartNavigator: a 3D application for real-time intra-operative guidance of device placement **(transcatheter aortic valve implantation).**<br>www.philips.com/a-w/about/news/archive/blogs/innovation-matters/20181112-how-a-virtual-heart-could-save-your-real-one.html
Siemens Healthineers	Digital twins of 100 patients with **congestive heart failure** were created and compared with outcome data of the physical twins over a six-year trial.<br>www.siemens-healthineers.com/en-au/news/mso-digital-gipfel.html
Sim & Cure	Simulation software to aide deployment of endovascular devices into an **intracranial aneurysm.**<br>www.sim-and-cure.com/product
